# Downregulation of RND3/RhoE in glioblastoma patients promotes tumorigenesis through augmentation of notch transcriptional complex activity

**DOI:** 10.1002/cam4.484

**Published:** 2015-06-24

**Authors:** Baohui Liu, Xi Lin, Xiangsheng Yang, Huimin Dong, Xiaojing Yue, Kelsey C Andrade, Zhentao Guo, Jian Yang, Liquan Wu, Xiaonan Zhu, Shenqi Zhang, Daofeng Tian, Junmin Wang, Qiang Cai, Qizuan Chen, Shanping Mao, Qianxue Chen, Jiang Chang

**Affiliations:** 1 Department of Neurosurgery, Renmin Hospital of Wuhan UniversityWuhan, Hubei, 430060, China; 2 Center for Translational Cancer Research, Institute of Biosciences and Technology, Texas A&M University Health Science CenterHouston, Texas, 77030; 3 Department of Neurology, Renmin Hospital of Wuhan UniversityWuhan, Hubei, 430060, China

**Keywords:** GBM, notch signaling, RND3, ubiquitination

## Abstract

Activation of Notch signaling contributes to glioblastoma multiform (GBM) tumorigenesis. However, the molecular mechanism that promotes the Notch signaling augmentation during GBM genesis remains largely unknown. Identification of new factors that regulate Notch signaling is critical for tumor treatment. The expression levels of RND3 and its clinical implication were analyzed in GBM patients. Identification of RND3 as a novel factor in GBM genesis was demonstrated in vitro by cell experiments and in vivo by a GBM xenograft model. We found that RND3 expression was significantly decreased in human glioblastoma. The levels of RND3 expression were inversely correlated with Notch activity, tumor size, and tumor cell proliferation, and positively correlated with patient survival time. We demonstrated that RND3 functioned as an endogenous repressor of the Notch transcriptional complex. RND3 physically interacted with NICD, CSL, and MAML1, the Notch transcriptional complex factors, promoted NICD ubiquitination, and facilitated the degradation of these cofactor proteins. We further revealed that RND3 facilitated the binding of NICD to FBW7, a ubiquitin ligase, and consequently enhanced NICD protein degradation. Therefore, Notch transcriptional activity was inhibited. Forced expression of RND3 repressed Notch signaling, which led to the inhibition of glioblastoma cell proliferation in vitro and tumor growth in the xenograft mice in vivo. Downregulation of RND3, however, enhanced Notch signaling activity, and subsequently promoted glioma cell proliferation. Inhibition of Notch activity abolished RND3 deficiency-mediated GBM cell proliferation. We conclude that downregulation of RND3 is responsible for the enhancement of Notch activity that promotes glioblastoma genesis.

## Introduction

Gliomas, tumors from glial origin, are the most prevalent primary tumors in the brain. Glioblastoma multiforme (GBM, World Health Organization grade IV), the highest grade of gliomas, are the most common malignant primary brain tumors with rapid and invasive growth, and fast development of resistance to chemoradiotherapy. Despite significant advances in neurosurgery and multiple therapeutic strategies, the median overall survival of glioblastoma patients remains just over 1 year 1. There is a critical need for new molecular targets, concepts, and approaches to treat this devastating disease. Notch signaling plays a critical role in the epidermal growth factor (EGF)/EGF receptor (EGFR) signaling amplification in GBM genesis 2,3. Hyperactivation of Notch has been observed in human GBM tissues and glioma cell studies 2,4,5. However, how the Notch signaling is augmented during GBM genesis remains largely unknown. In this study we revealed RND3 (also called RhoE), a small GTPase, as an endogenous inhibitor of Notch signaling. Downregulation of RND3 detected in human GBM was responsible for the hyperactivation of Notch signaling, which promotes gliomagenesis.

RND3 is an atypical member of the Rho GTPase family in that it lacks detectable GTPase activity. The best characterized functions of RND3 are its inhibitory effect on Rho kinase-mediated biological functions including actin cytoskeleton formation, phosphorylation of myosin light chain phosphatase, and apoptosis 6–8. Two recent mouse studies revealed an indispensable role of RND3 in mouse neuron development 9,10. Using a mouse genetic approach, we showed that RND3 is highly expressed in mouse brain tissues. RND3 deficiency promotes mouse ependymal epithelia proliferation, which in turn results in aqueduct stenosis and hydrocephalus development 11. However, the pathological role of RND3 in human GBM progression and the associated animal studies has not been investigated.

Here, we provide evidence to show that the expression levels of RND3 are significantly downregulated in human glioblastomas. RND3 protein expression levels are inversely associated with tumor size, tumor cell proliferation, and Notch activity, and positively correlated with patient survival time. We demonstrate that RND3 physically interacts with Notch transcriptional complex cofactors and facilitates their protein degradation, which prevents hyperactivation of Notch signaling. Overexpression of RND3 diminishes Notch signaling, which in turn inhibits glioma cell proliferation and tumor growth in xenograft mice. Downregulation of RND3 enhances Notch signaling activity, promoting glioma cell proliferation. Finally, the inhibition of Notch activity by chemical compound E treatment or by siRNA specific for Notch1 application abolishes RND3 deficiency-mediated GBM cell proliferation. Our findings reveal a previously undescribed function of RND3 in GBM genesis and provide a new insight into the inhibitory effect of RND3 on the Notch activation regulation mechanism. Importantly, the discovery gives a mechanistic explanation of how the Notch activity is augmented in gliomagenesis.

## Materials and Methods

### Human GBM samples and control brain tissues

Human GBM (grade IV) tissues were obtained at the time of surgery from the Department of Neurosurgery and Neurology in Renmin Hospital of Wuhan University. Control non-GBM adult human brain tissues were collected from unmatched patients undergoing surgery for intracranial hypertension. Tumor histology diagnosis was confirmed independently by two neuropathologists. The procurement of tissue usage for the study was obtained with written patient-informed consent and approval by the Institutional Ethics Committee of the Faculty of Medicine at Renmin Hospital of Wuhan University (approval number: 2012LKSZ(010)H). The detailed demographics of patients were presented in Tables S1 and S2.

### Generation of human orthotopic GBM xenograft mouse model

Congenitally athymic male nude mice, 5–8 weeks old (Charles River Laboratories, Wilmington, MA), were used. Intracranial injection of U251 cells into the lateral ventricle was performed as described previously 12. In summary, the mice were anesthetized by isoflurane, and a 1 cm sterile midline scalpel was prepared for a sagittal incision over the parieto-occipital bone. A sterile 25 gauge needle was used to penetrate into the skull at the position 2 mm to the right of the bregma and 1 mm anterior to the coronal suture. A total concentration of 5 × 10^5^ cells in 3 *μ*L of PBS, or PBS alone for the control, were injected slowly. Tumor growth was analyzed 15 weeks after the injection surgery. All experiments with animals were approved by the Institutional Animal Care and Use Committee of the Texas A&M University Health Science Center-Houston.

### Cell culture, gene transient transfection, generation of stable cell lines, and BrdU staining analysis

Human malignant glioma (U251 and U87) cells were generously provided by Dr. Zhimin Lu (MD Anderson Cancer Center), and were cultured in Dulbecco's Modified Eagle's Medium (DMEM) with 10% FBS (fetal bovine serum). All of the gene transient transfections were conducted by the NEON transfection system (MPK5000; Life Technologies Carlsbad, CA, USA). For bromodeoxyuridine (BrdU) staining analysis, cells were incubated with BrdU for 45 min before being harvested. The images were acquired by fluorescence microscopy.

The siRNA pools containing multiple siRNAs specific for RND3, NOTCH1, and nontargeting siRNAs as a control were purchased from Thermo Scientific Dharmacon RNAi technologies (RND3: L-007794-00-0005; NOTCH1: E-007771-00-0005, nontargeting siRNA: D-001206-13-20; Lafayette, CO). Construction of myc-RND3 and Flag-NICD (Notch intracellular domain) overexpression vectors were described previously 11. Myc-FBW7 (F-box and WD repeat domain-containing 7) (pCMV-Myc CDC4 R465C) and HA-Ubiquitin expression vector were purchased from Addgene. Flag-NICD, myc-CSL (CBF1-suppressor of hairless-Lag-1) and Flag-MAML1 (mastermind-like protein 1) were generously provided by Dr. Chundong Yu (Xiamen University).

To establish the RND3 knockdown stable cell line and RND3 constitutive expression stable cell line, lentiviral vectors V3LHS_346799 (Thermo Scientific) expressing short hairpin RNAs specific for human RND3, and pLVX-AcGFP1-C1 Vector (632155, Clontech Mountain View, CA, USA) with insertion of human RND3 cDNA were used, respectively, in U251 cells. As the controls, a vector expressing nonsilencing shRNA (RHS4346) and pLVX-AcGFP1-C1 vector without RND3 cDNA insertion were used. Briefly, the 293FT (Invitrogen, Carlsbad, CA) cells were transfected with the lentiviral vector expressing specific shRNA with two helper vectors, pMD2.G and psPAX2, to produce the lentivirus. Cells were infected with the lentiviral virus at an multiplicity of infection (MOI) of 10 with Polybrene (8 g/mL) to enhance the virus transduction. The efficiency of viral infection was monitored by GFP expression. Puromycin (10 g/mL) was used for the cell selection 13.

Compound E, (2S)-2-(((3,5-Difluorophenyl)acetyl)amino)-*N*-((3S)-1-methyl-2-oxo-5-phenyl-2,3-dihydro-1H-1,4-benzodiazepin-3-yl), (AG-CR1-0081; Adipogen, San Diego, CA, USA), was dissolved in DMSO (dimethyl sulfoxide) with a final concentration of 1 *μ*mol/L for cell culture studies. The concentration of DMSO was ≤0.1% in media. U251 cells were harvested after 4 h treatment with MG132 at 10 *μ*mol/L. A final concentration of 50 *μ*g/mL cycloheximide was applied for the cycloheximide chase experiment for 15 h.

### QPCR analysis

Messenger RNAs were quantified by quantitative PCR (qPCR) analysis (Applied Biosystems StepOnePlus (Carlsbad, CA, USA)) using the SYBR green method with a MasterMix buffer system containing Taq polymerase (Stratagene, USA) as described previously 15. Total RNA was prepared by TRIzol extraction (Gibco BRL). The forward and reverse PCR primers (5’ to 3’): HES1 (hairy and enhancer of split-1): CGGACATTCTGGAAATGACA/CATTGATCTGGG TCATGCAG; RND3: CTATGACCA GGGGGCAAATA/TC TTCGCTTTGTCCTTTCGT; NOTCH1: CGGACATTCT GGAAATGACA/CATTGATC TGGGTCATGCA; CSL: CGC ATTATTGGATGCAGATG/CAGGAAGCGCCATCA TTTAT; MAML1: CAGCATCAGTTGCTTTTGGA/CCC TGTGAACTGTCCAACCT; GAPDH: GAGTCAACGGAT TTGGTCGT/TTGATTTTGGAGGGATCTCG. GAPDH expression levels were used for qPCR normalization. Expression levels were determined by the 2^−ΔΔCt^ threshold cycle method.

### Immunostaining, immunoblotting, and immunoprecipitation

The following antibodies were used for immunoanalyses: anti-RND3 (Cocalico Biologicals, Reamstown, PA, USA), anti-NOTCH1 (Abcam, Boston, MA, USA ab27526), anti-HES1 (Abcam, ab71559), anti-pHis3 (Santa Cruz, Dallas, TX, USA sc-8656), anti-c-Myc (9E10, Santa Cruz, sc-40), anti-Flag (sigma, St. Louis, MO, USA F7425), anti-Histone H3 (Rabbit, abcam, ab1791), anti-GFP (Rabbit, Santa Cruz, sc-5385), anti-LaminB (Rabbit, Santa Cruz, sc-20682), APC-conjugated anti-BrdU antibody (BD, 552598). The specificity and sensitivity of the anti-RND3 antibody was validated in our previous study 14. Even protein loading for immunoblotting analysis was verified by the intensity of the GAPDH blot (Santa Cruz, sc-20357). The immunoblotting densitometry was quantified by the Gel Logic 6000 PRO Imaging System (Carestream Health, Inc. Rochester, NY, USA), and the immunofluorescent and immunohistochemical image quantifications were conducted by Leica Application Suite Imaging Software (Version 4.0, Biberach, Germany).

### Luciferase assay

Luciferase reporter vector with a promoter containing four CSL-binding elements was purchased from Addgene (Cambridge, MA, USA) (pCBFRE-luc, plasmid#26897). The luciferase assay was conducted 36 h posttransient transfection. Lysis buffer was purchased from Promega (Madison, WI, USA) (E3971), and 20 of 150 *μ*L of the lysis supernatant were used with a Lumat3 LB9508 luminometer (Berthold, Oak Ridge, TN, USA) for the measurement of luciferase activity. Each sample was measured three times. All results were normalized to *β*-galactosidase activity (MRX Revelation; DYNEX Technologies, CHANTILLY, VA, USA).

### Quantitative ChIP assay

To define the regulation of RND3 with NOTCH1-related transcription activity in vivo, multiple ChIP assays were performed as described previously using the Abcam ChIP kit (ab500, UK) 15. Cells were gently fixed with formaldehyde and lysed by sonication 36 h after the transient transfection. The specific NICD-DNA complex was immunoprecipitated using anti-NOTCH1 antibody. The identity of the CSL-binding elements from *HES1* isolated from the complex with NICD was determined and quantified by qPCR, and the results were normalized with each histone pull-down Ct value. The primers (5’ to 3’) for the detection of fragments were: CGTGTCTCCTCCTCC CATT/GGCCTCTATATATATCTGGGACTGC, and the final PCR products were then sequenced to confirm the presence of the CSL-binding elements.

### Statistics

Data were expressed as means ± standard errors of the means. Statistical analysis was performed with (version 11.0; SigmaPlot and 13.0; SPSS San Jose, CA, USA). Differences between means were assessed with the student's *t*-test, paired *t*-test, or Mann–Whitney *U*-test for abnormally distributed data. In multiple comparisons, one-way analysis of variance (ANOVA) was used. Pearson's test was used to detect the correction of two groups and compare quantitative values of expression. Survival curves were plotted by the Kaplan–Meier method and compared by log-rank test. A value of *P* < 0.05 was considered statistically significant.

## Results

### RND3 expression was reduced in human glioblastoma

**Figure 1:**
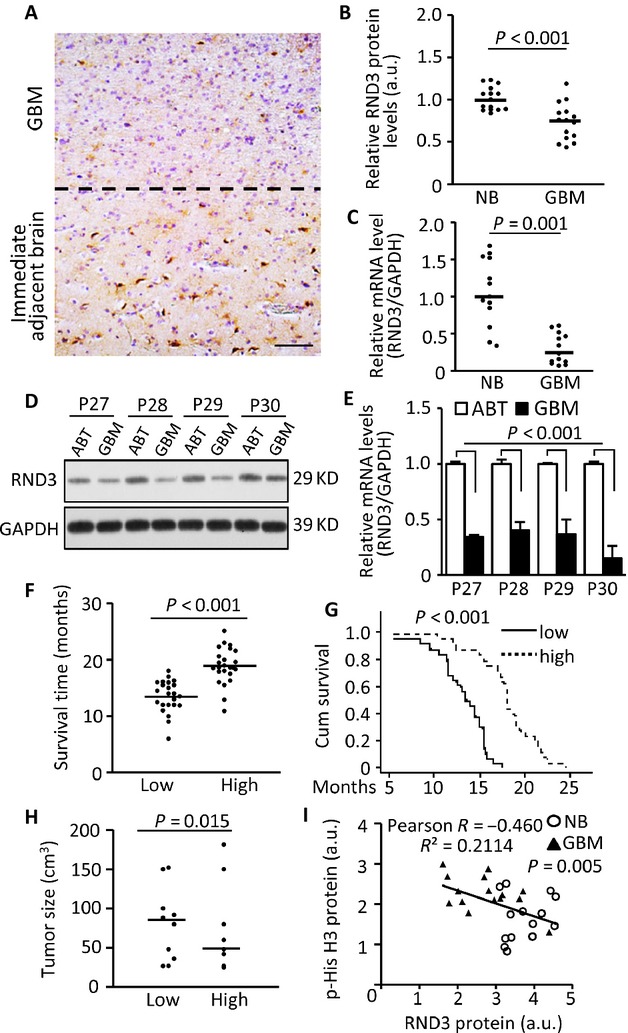
RND3 expression levels were downregulated in human glioblastomas, and were correlated with glioblastoma multiform (GBM) patient survival, and inversely associated with tumor size, and tumor cell proliferation. (A) Representative immunohistochemical staining displayed a reduced staining for RND3 (brown) in human glioblastoma tissue (GBM) compared to the immediate adjacent brain tissue (ABT). The dotted line shows the boundary between the GBM and the ABT. Blue color indicates nuclear staining. (B) Quantification of the immunohistochemical staining for RND3 in patients with GBM (*n* = 15) and normal brain (NB) tissues (*n* = 15). (C) Significant decreases in RND3 mRNA levels in GBM patients (*n* = 13) compared to NB tissues (*n* = 13) were detected by qPCR analysis. (D and E) Paired analyses between GBM and the coordinated ABT in RND3 protein and transcript levels showed the downregulation of RND3 in GBM. P27 to P30 indicates patients 27–30 (Table S1). (F and G) Patients with lower RND3 protein levels showed shorter survival times compared to the patients with higher RND3 protein levels. (*n* = 25 in low levels of RND3 group, *n* = 24 in high levels of RND3 group). Patient survival times were measured from postsurgery to death. (H) Comparison of tumor size and the associated RND3 protein expression levels (*n* = 10 in each group). Tumor size was assessed immediately after surgery. (I) An inverse correlation between the phosphorylated histone H3 (p-His H3) levels and the RND3 protein levels was shown when GBM tissues (*n* = 15) were compared to NB tissues (*n* = 15). *P*-values from the Mann–Whitney *U*-test (B, F, and H), the Student's *t*-test (C), paired *t-*test (E), and the Kaplan–Meier test (G). Statistical analysis of correlation was performed with Pearson's test (I). Scale bar, 50 *μ*m. a.u., arbitrary unit.

To determine the clinical significance of RND3 in gliomas, we assessed and compared the expression levels of RND3 transcript and protein in human GBM specimens, the glioma adjacent brain areas (tissues) (ABT, 3 cm away from glioma), and normal brain tissues (Table S1). A representative immunohistochemical staining exhibited reduced RND3 protein expression in human GBM tissue compared to the immediate ABT (Fig.1A). We quantified the immunohistochemical staining for RND3 in 15 human GBM specimens and 15 human normal brain tissues, and found a significant reduction of RND3 expression in the GBM specimens compared to the normal brains (Fig.1B). Consistent with the RND3 immunohistochemical staining assessments, RND3 mRNA levels were decreased in GBM tissues when compared to normal brain tissues (Fig.1C). To further verify the downregulation of RND3 expression in GBM, we compared the expression levels of RND3 protein (Fig.1D) and transcript (Fig.1E) in four pairs of patient-matched GBM specimens and the corresponding glioma ABTs. Both western blot and qPCR analysis showed the clear decline of RND3 expression in the GBM specimens.

### RND3 protein expression levels were positively correlated with GBM patient survival, but inversely associated with tumor size and tumor cell proliferation

To explore the relationship of RND3 expression levels and glioblastoma progression, we divided GBM patients into two groups: low and high expression levels of RND3 determined by quantitative image analysis with densitometry for immunohistochemistry. The median of the RND3 densitometries was used to divide the patients into the two groups, with 24 patients in the high group and 25 in the low group (Table S2).

We then compared the two groups of patients based on survival times and tumor sizes. As shown in Figure1F and G, patients with a high expression level of RND3 had a significantly longer survival time compared to the patients with a low expression level of RND3. The average tumor size was larger in patients with low expression of RND3 compared to the patients with high expression of RND3 although the tumor sizes were variable (Fig.1H). To confirm the inverse correlation between RND3 protein expression levels and GBM tumor growth, we detected phosphorylated histone 3 (p-His3) levels, a cell proliferation marker, in GBM and human normal brain tissues by western blot analysis. Consistently, GBM tissues showed lower expression levels of RND3 along with higher levels of p-His3 compared to normal brains (Fig.1I); suggesting an inverse correlation between RND3 expression levels and GBM progression.

### Forced expression of RND3 inhibited glioblastoma cell proliferation and GBM tumor growth in mice

**Figure 2:**
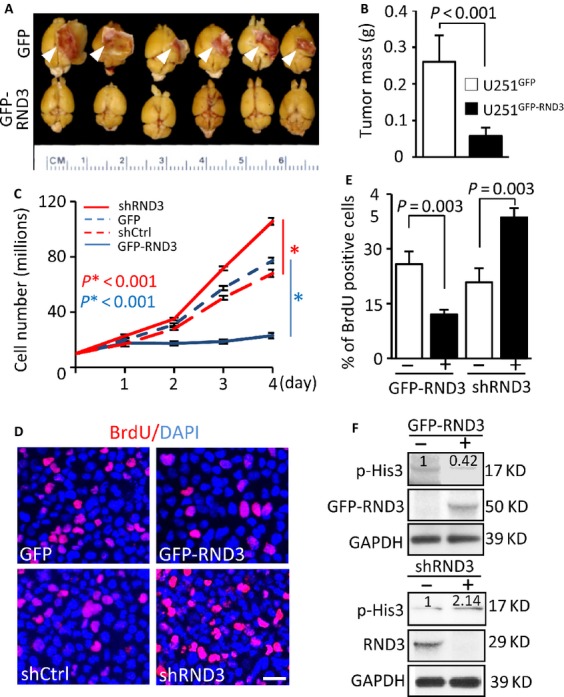
Forced expression of RND3 inhibited glioblastoma cell proliferation and glioblastoma multiform (GBM) growth in mice. (A and B) GBM growth was significantly hindered in nude mice with intracranial implantation of GFP-RND3 cells, a human glioblastoma cell line U251 with GFP-RND3 stable expression, compared to the control mice with intracranial implantation of U251 cells expressing GFP (*n* = 9 in each group). (C–F) Two U251 cell lines, GFP-RND3 stable expression cell line and RND3 knockdown cell line by shRND3, were synchronized to assess the cell growth rate. In the GFP-RND3 stable expression line, a retarded growth rate (C, solid blue line) was observed with fewer BrdU-stained cells (D and E) and lower p-His3 levels (F, top panel). The number at the top of each band represents the average of densitometries from three experiments, normalized by GAPDH. An opposite result was detected in RND3 knockdown cells. The data of each time point in panel C represent the average of three measurements. In panel E, BrdU-positive cells were quantified from nine images taken from three slides. Data represent means ± SE. Scale bar represents 25 *μ*m. GFP-RND3 molecular weight is around 50 kD, while the endogenous RND3 molecular weight is about 29 kD. *P*-values were from Student's *t*-test (B) and one-way ANOVA test (C and E).

To determine the inhibitory role of RND3 in gliomagenesis, we generated a human orthotopic GBM xenograft animal model by intracranial implantation of U251 glioblastoma cells in nude mice. As expected, mice with the implantation of GFP-tagged U251 cells developed massive GBMs (Fig.2A). All of the tumors broke the cerebral cortex 15 weeks after implantation. However, in the mice implanted with the RND3 stable expression U251 cell line (GFP-RND3), none of the animals developed tumors that invaded beyond the cerebral cortex (Fig.2A). The tumor size developed from the RND3 stable expression U251 cells was 4-folds smaller than the tumors developed from the GFP-tagged U251 cells (Fig.2B).

To assess RND3-mediated U251 cell growth retardation, equal amounts of cells were synchronized and cultured in a growth medium to quantify the cell growth rate. As shown in Figure2C, the cell growth was significantly impeded in the RND3 stable expression cell line (solid blue line) while the cell growth was facilitated in the RND3 knockdown cell line (solid red line). Meanwhile, this RND3-mediated cell proliferation regulation was further confirmed by BrdU pulse-labeling at a 12 h time point (Fig.2D). The number of BrdU-positive cells in the RND3 stable expression cell line was about half of the control level (Fig.2E). The opposite result was detected in the cells with RND3 knockdown, in which downregulation of RND3 doubled the BrdU-positive cells compared to the control (Fig.2D–E). To analyze the cell proliferation status under RND3 overexpression and downregulation conditions, we measured p-His3 levels. Consistent with the xenograft, the cell growth rate, and the BrdU labeling assay, forced expression of RND3 attenuated the p-His3 levels and downregulation of RND3 promoted the p-His3 levels (Fig.2F). The inhibitory effect of RND3 on glioblastoma cell proliferation was further confirmed in an alternative glioblastoma cell line, U87 (Fig. S1).

### RND3 inhibited Notch signaling activity

**Figure 3:**
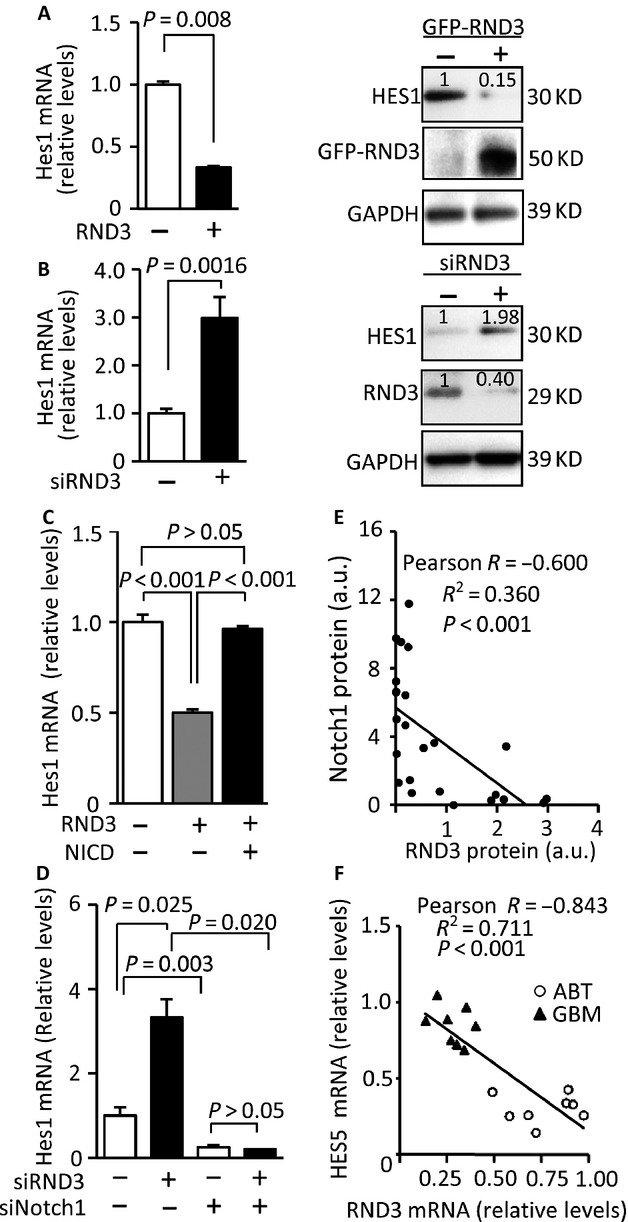
RND3 negatively regulated Notch signaling, and the expression levels of RND3 were inversely correlated with Notch signaling in human GBM tissues. (A) Forced expression of RND3 inhibited NOTCH1 target HES1 transcript (left panel) and protein levels (right panel) in U251 cells. (B) Knockdown of RND3 resulted in upregulation of HES1 transcript (left panel) and protein levels (right panel) in U251 cells. The number at the top of each band represents the average of densitometries from three experiments, normalized by GAPDH. (C) Forced expression of Notch active isoform NICD diminished RND3-mediated HES1 downregulation. (D) Knockdown of NOTCH1 abolished RND3 deficiency-induced upregulation of HES1. (E) Quantification of the immunohistochemical staining for RND3 and NOTCH1 showed an inverse correlation of the two protein expression levels in human GBM tissues (*n* = 30). (F) An inverse correlation between the HES5 transcript levels and the RND3 transcript levels was shown when GBM tissues (*n* = 8, patient 23–30) were compared to the corresponding tumor adjacent brain tissues (ABT). Statistical analysis of correlation was performed with Pearson's test (E and F).

We recently demonstrated that RND3 functions as an endogenous inhibitor of Notch in mouse ependymal epithelia. Genetic deletion of *Rnd3* resulted in aqueductal stenosis in mouse brains through upregulation of Notch signaling 11. We wished to test if RND3 regulates Notch activity in human GBM cells. The transcript and protein levels of *HES1*, a Notch direct target gene, were assessed under RND3 overexpression and knockdown conditions. Forced expression of RND3 in U251 cells significantly inhibited HES1 transcript and protein expression (Fig.3A), while the knockdown of RND3 enhanced HES1 transcript and protein expression (Fig.3B). The negative regulation of RND3 on HES1 was also detected in U87 glioblastoma cells (Fig. S2 A and B). To test if RND3-mediated HES1 regulation is directly associated with Notch activity, we introduced Notch active isoform NICD into cells and found that forced expression of NICD overcame RND3-induced HES1 downregulation (Fig.3C). Meanwhile, the knockdown of Notch completely abolished RND3 deficiency-induced HES1 upregulation (Fig.3D). Both data strongly suggest that RND3 negatively regulates Notch signaling in human glioma cells.

### RND3 expression levels were inversely correlated with Notch signaling in human GBM tissues

To extend the clinical investigation of RND3 and Notch activity, we then evaluated the correlation of RND3 expression levels and Notch activity in 30 human GBM specimens. RND3 protein expression levels and the corresponding NOTCH1 protein expression levels were assessed by immunohistochemical staining. The data indicated an inverse correlation between RND3 protein levels and NOTCH1 protein levels with a −0.6003 Pearson product-moment correlation coefficient (Fig.3E). To further validate this close relationship between RND3 and Notch activity in human GBMs, we quantified the transcript levels of Notch target gene *HES5*, a major isoform of HES family members in humans, and compared the levels of HES5 transcript in GBMs to the levels in the tumor adjacent brain regions. As shown in Figure3F, all of the eight GBM tissues displayed higher expression levels of HES5 mRNA along with lower levels of RND3 mRNA compared to the corresponding tumor ABTs, indicating a strong negative correlation between RND3 expression and HES5 transcript levels.

### RND3 physically interacted with Notch transcriptional complex, NICD-CSL-MAML1 component. Forced expression of RND3 promoted Notch complex factor protein degradation through enhancement of FBW7-mediated NICD ubiquitination

**Figure 4:**
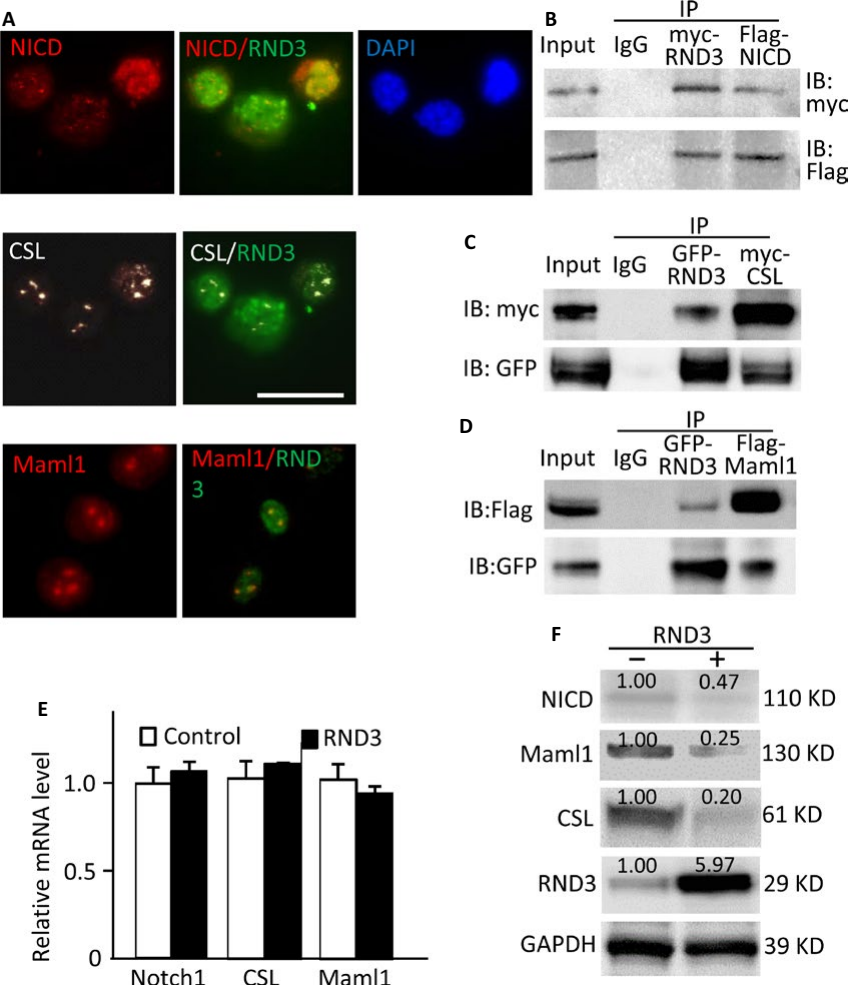
RND3 physically interacted with NICD-CSL-MAML1 complex and facilitated its degradation. (A) Immunofluorescent staining displayed the colocalization of endogenous RND3 with NICD (upper panel), CSL (middle panel), and MAML1 (lower panel) in U251 glioblastoma cells. (B-D) Coimmunoprecipitation (IP) pull-downs were performed, followed by immunoblotting analyses (IB). The blots confirmed the interactions of RND3 with NICD, CSL, and MAML1. (E) qPCR analysis indicated no changes in NOTCH1, CSL, and MAML1 transcript levels by forced expression of RND3. The data were pooled by three experiments, with analysis for each in triplicate. (F) Immunoblotting analyses showed that forced expression of RND3 resulted in decreases in NICD, MAML1 and CSL protein levels in U251 glioblastoma cells. The number at the top of each band represents the average of densitometries from three experiments, normalized by GAPDH. Scale bar represents 20 *μ*m.

To reveal the molecular mechanism of RND3-mediated Notch signaling repression, we assessed the interactions of RND3 with three key cofactors in Notch transcriptional complex, NICD, CLS, and MAML1. The colocalization of endogenous RND3 with NICD, CSL, and MAML1 were visualized in U251 glioblastoma cells by immunofluorescent staining (Fig.4A). We then performed coimmunoprecipitation pull-down assays, followed by immunoblotting analyses. The blots confirmed the interactions of RND3 with NICD (Fig.4B), CSL (Fig.4C), and MAML1 (Fig.4D) in vivo. Since forced expression of RND3 inhibited Notch activity, we evaluated the transcript and protein levels of the three cofactors when RND3 was overexpressed in cells. qPCR analysis indicated no changes in NOTCH1, CSL, or MAML1 transcript levels by forced expression of RND3 (Fig.4E). However, NICD, MAML1, and CSL protein levels were significantly decreased in U251 glioblastoma cells with RND3 overexpression (Fig.4F).

**Figure 5:**
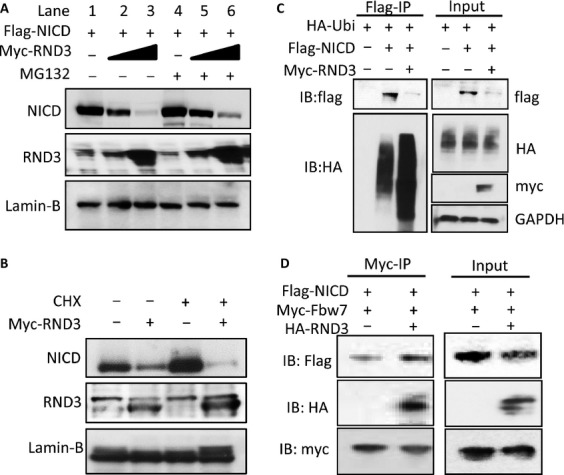
RND3 promoted NICD ubiquitination and protein degradation by facilitating NICD-FBW7 interaction. (A) Noticeable decreases in NICD protein levels were observed along with the elevated RND3 protein levels; this effect was partially attenuated by the treatment with a proteasome inhibitor, MG132. (B) Immunoblot analysis showed that the degradation of endogenous NICD was enhanced by the introduction of RND3 even when protein synthesis was inhibited by the treatment of cycloheximide (CHX). (C) A representative immunoblotting analysis for ubiquitin showed the increase in the ubiquitination level of NICD pull-down complex in the forced expression of RND3 group. Meanwhile, the reduction in NICD protein levels was detected in parallel with this enhanced ubiquitination. (D) With forced expression of RND3, more FBW7-bound NICD protein was detected (Myc-IP section). As a result, less total NICD protein was observed in the same pull-down sample (Input section).

Ubiquitination is a critical posttranslational regulatory mechanism for NICD complex degradation. Since NICD is a central molecule of the NICD complex, we further validated the change in NICD protein levels along with the increase in RND3 expression after the treatment of MG132, a proteasome inhibitor. Again, the immunoblot analysis showed the decline of NICD protein levels when the cells expressed higher levels of RND3 (Fig.5A, lanes 1-3). However, we observed attenuation of NICD degradation as early as 4 hours after MG132 treatment (Fig.5A, lanes 4–6). To rule out that the RND3-induced NICD protein level decline was not due to weakening protein synthesis, we conducted a cycloheximide (CHX) chase analysis with the protein synthesis inhibitor, CHX. The Rnd3-induced NICD degradation was confirmed in the CHX chase analysis. The lower endogenous NICD protein levels were observed in cells transfected with the RND3 expression vector compared to the control cells (Fig.5B).

Then, we evaluated the levels of NICD ubiquitination by immunoprecipitation of NICD followed by anti-ubiquitin immunoblotting analysis. As shown in Figure5C (lower panel in the Flag-IP section), the amount of ubiquinated species was obviously increased by the addition of RND3. In parallel with the increase in NICD ubiquitination, the correlated lower expression levels of NICD protein were detected in the same pull-down sample (Fig.5C, upper panel in the Flag-IP section). The control and even loading were validated and shown in the Input section.

Finally, we wanted to determine how RND3 promoted NICD degradation. We assessed the amount of NICD that was bound to FBW7, a ubiquitin ligase for NICD, by an immunoprecipitation assay. We found that more NICD protein was pulled down by FBW7 in the RND3 transfected group compared to the control (Fig.5D, the Myc-IP section), suggesting that RND3 facilitated the interaction of NICD with its ubiquitin ligase, FBW7. In the same cohort of samples, a coordinated decrease in the NICD protein levels was once again detected in the RND3 transfected group (Fig.5D, the Input section). These data provide evidence that RND3 is a regulatory factor that interacts with NICD complex and promotes ubiquitin-proteasome system (UPS)-mediated NICD complex degradation by promoting the binding of NICD to its ubiquitin ligase, FBW7.

### NOTCH1 transcriptional activity was regulated by RND3

**Figure 6:**
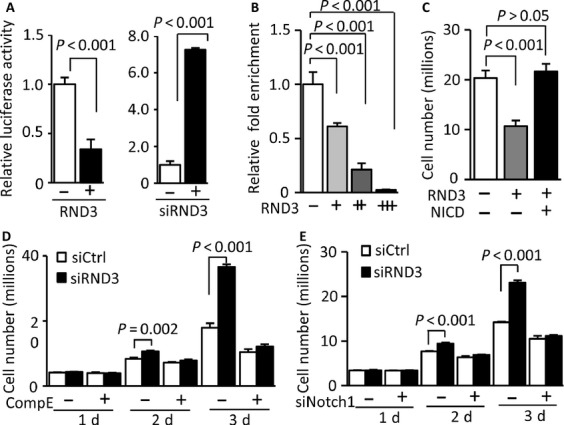
NOTCH1 transcriptional activity was regulated by RND3, and inhibition of Notch activity diminished RND3 deficiency-induced GBM cell proliferation. (A) Addition of RND3 inhibited luciferase activity driven by CSL-responsive elements (left panel); and knockdown of RND3 promoted the luciferase activity (right panel). (B) Quantitative chromatin immunoprecipitation (ChIP) assay showed that NICD transcription-binding activity was significantly diminished along with the increase in RND3 expression levels in the *HES1* promoter region. (C) Forced expression of RND3 inhibited U251 cell growth and the addition of NICD abolished the inhibitory effect of RND3 on cell proliferation. (D) Knockdown of RND3 promoted cell growth and compound E (Comp E) treatment impeded RND3 deficiency-induced cell proliferation. (E) Knockdown of *NOTCH1* by siRNA (siNotch1) also abolished RND3 deficiency-induced cell proliferation.

To further confirm the functional effect of RND3 on Notch transcriptional activity, a duplex containing multiple *CSL*-responsive elements was subcloned into a luciferase reporter vector. The effect of RND3 on the luciferase expression driven by the duplex was detected from the changes in luciferase activity. We found that cotransfection of the reporter and RND3 expressing vectors led to a 66% reduction in luciferase activity compared to the baseline control without RND3 coexpression (Fig.6A, left panel) in U251 glioblastoma cells. In a parallel experiment, the knockdown of RND3 resulted in a >7-fold increase in luciferase activity compared to the baseline control (Fig.6A, right panel), indicating negative transcriptional regulation of Notch transcriptional activity by RND3. The experiments were also conducted in U87 glioblastoma cells and the same result was observed (Fig. S2C and D).

To verify that RND3 interacts with the endogenous *HES1* promoter through the Notch regulatory elements in vivo, a series of quantitative chromatin immunoprecipitation (ChIP) assays were performed 16. The specific RND3-Notch complex was immunoprecipitated using an anti-RND3 antibody. As shown in Figure6B, the overexpression of RND3 resulted in a dramatic decrease in the Notch-binding nucleotides pulled down from the cells, and this decrease was furthered when RND3 expression was increased.

### Notch activation was responsible for RND3-regulated GBM cell proliferation and inhibition of Notch activity diminished RND3 deficiency-induced GBM cell growth

We have provided evidence that forced expression of RND3 inhibited GBM cell growth (Fig.2C) and repressed Notch transcriptional activity (Fig.6A and B). To understand the biological relationship between RND3 expression and Notch activation, we investigated if manipulation of Notch activity would have any effects on RND3-induced GBM cell proliferation. As shown in Figure6C, introduction of NICD abolished RND3-induced cell growth inhibition. We then conducted experiments using compound E, a Notch signaling inhibitor. We found again that the knockdown of RND3 promoted U251 cell growth and this increase was completely impeded by the compound E treatment (Fig.6D). To verify the result, the same experiments were performed using a specific siRNA for the knockdown of *NOTCH1*. Again, RND3 deficiency-induced cell proliferation was abolished by the knockdown of *NOTCH1* (Fig.6E). The result indicates that Notch activation is responsible for RND3 deficiency-induced glioma cell proliferation.

## Discussion

The direct and mechanistic role of RND3 in GBM tumorigenesis remains largely unexplored. The expression levels of RND3 have not been associated with human GBM. The current paradigm, derived from the majority of the published studies on RND3, is that RND3 functions as an inhibitor of Rho signaling by either directly binding to ROCK1 or indirectly targeting RhoA through p190-B RhoGAP 6,7,17,18. Overexpression of RND3 inhibited ROCK1-mediated biological effects, and the reduced expression of RND3 stimulated ROCK1 activity 6–8. Therefore, the functions of RND3 have been almost entirely linked to cell actin cytoskeleton dynamics, cell migration, and apoptosis demonstrated by in vitro cell culture studies 6,7,19,20.

Emerging evidence starts to reveal that RND3 participates in multiple forms of biological functions that are likely independent of Rho kinase activity 11,21. In one cell culture study, Villalonga et al. observed that the forced expression of RND3 inhibited NIH 3T3 fibroblast proliferation and this RND3-mediated G_1_ cell cycle arrest was not associated with RhoA/ROCK signaling activation, but was due to the decreased expression level of cyclin D1 protein along with the inhibition of pRb pocket protein 21. A similar observation was made in glioma cells 22. We recently reported that the mice with *Rnd3* genetic deletion developed severe hydrocephalus, a mouse brain phenotype that was not found in any reported Rho kinase mutant mice 3,15,23,24. The indispensable role of Rnd3 in mouse brain development was also observed in two recent studies 9,10. Given the importance of Rnd3 in mouse brain physiology and the abundance of Rnd3 expression in the brain, we proceeded to investigate if RND3 was involved in brain GBM tumorigenesis. In the analysis of human GBM biopsies, we detected significant declines in RND3 transcript and protein levels in GBM tissues compared to normal human brain tissues and even in the corresponding tumor ABTs. With limited clinical epidemiology assessments, we found that the RND3 protein levels were closely correlated with GBM patient survival times, but inversely associated with patient tumor size and tumor cell proliferation. The mouse xenograft studies further indicated that ectopic expression of RND3 dramatically hindered the tumor growth. Consistent with the xenograft mouse study, forced expression of RND3 inhibited human glioblastoma cell proliferation, while RND3 knockdown facilitated glioblastoma cell growth in cell culture. Thus, several lines of evidence, from human patients to an in vivo mouse model and in vitro cell studies, strongly suggest an inhibitory role of RND3 in GBM tumorigenesis. Manipulation and assessment of RND3 expression levels could be a potential strategy for GBM treatment and prognosis, respectively.

Activation of the EGF/EGFR signaling pathway is the major proliferation mechanism in GBM genesis 1. In the cross-talk between EGF/EGFR and Notch signaling, NOTCH1 transcriptionally upregulates EGFR 25. Notch signaling-mediated genes are overexpressed in GBM with EGFR amplification 3, suggesting Notch signaling as an important activator of EGF/EGFR signaling amplification in GBM. It is generally believed that Notch signaling integrity is critical for maintaining undifferentiated progenitor cells from differentiating into neurons; and NOTCH expression is reduced in the adult central nervous system. The pro-oncogenic role of Notch activation in GBM is thought to reflect its biological function in normal CNS development. Hyperactivation of Notch signaling will inhibit progenitor cell differentiation and could promote GBM formation 2. In primary GBM patients, the significant increases in NOTCH1, Notch target HES1, and Notch ligands Jagged1 Delta-like 1 (DLL1) transcripts were detected in GBM biopsies 4,5. Knockdown of *NOTCH1* or its ligands inhibited GBM cell proliferation in cell and animal studies 5. Clearly, evidence from both clinical and basic research strongly implicates the association between the hyperactivation of Notch signaling and gliomagenesis. However, one of the important questions remains unresolved, which is how Notch signaling is augmented in primary GBM. In this study, we at least provided a partial answer to the question as we identified RND3 as an endogenous inhibitor of Notch in gliomas. We demonstrated that RND3 expression was significantly decreased in human GBM tissues, and the downregulation of RND3 was partially responsible for the increased Notch activation. The RND3 deficiency-mediated glioma cell aberrant proliferation can be blocked by Notch inhibitor compound E treatment or by the knockdown of *NOTCH1*. Moreover, we found that the expression levels of RND3 were inversely correlated with Notch activities in human GBM tissues.

The mechanism of RND3-mediated Notch regulation was first revealed by our early RND3 knockout mouse study 11. We demonstrated that RND3 physically interacts with NICD, the Notch active isoform, and titrates the NICD availability by mediating NICD protein ubiquitination. The deficiency of RND3 weakens the process and results in an increase in NICD levels in cells, augmenting Notch signaling in ependymal cells 11. In this study, we confirmed the inhibitory regulation of RND3 on Notch in glioma cells, and quantified the negative effects of RND3 on Notch activity by multiple quantitative ChIP assays. We validated NICD factor as an RND3 target in glioma cells.

Meanwhile, we want to indicate that there are three new mechanistic findings that extend our previous study. First, we identify that RND3 interacts with two other cofactors in the Notch transcriptional complex, CSL and MAML1. RND3 functions as an endogenous inhibitor of Notch. Binding of RND3 with these three factors prevents them from forming a functional complex, and facilitates the protein degradation of the three cofactors. Secondly, we reveal the molecular mechanism that RND3 can destabilize the Notch complex by facilitating NICD interaction with FBW7, the substrate recognition component of SCF (complex of SKP1, CUL1 and F-box protein)-type ubiquitin ligase that can degrade NICD 26. Lastly, we demonstrate that downregulation of RND3 results in the formation of more regulatory complexes that hyperactivate Notch signaling. The latter promotes glioma cell proliferation and tumor growth (Fig. S3).

Interestingly, while there was no new mechanism revealed, one recent study confirmed our previous *Rnd3* knockout mouse study and observed RND3-mediated Notch inhibition in non-small cell lung cancer cell lines 27. However, an opposite relationship between RND3 and Notch signaling was recently reported in squamous cell carcinomas 28. The study showed that the *RND3* gene was a transcriptional target of NOTCH1, and RND3 promoted Notch signaling by facilitating NICD nuclear translocation through importin *β*1 in skin epithelial cancer cells. The two different functions of RND3-medaited Notch regulation may indicate the importance of maintaining Notch signaling integrity, which requires both positive and negative regulation mechanisms. On one hand, when Notch activity is too high, RND3 will put a “brake” on the Notch regulatory complex; however, RND3 will promote Notch activity when its activation is too low. We believe that both mechanisms are necessary for normal Notch signaling. It should also be realized that, opposite to GBM, Notch signaling is downregulated in skin cancer 28. Given the fact that the decrease in RND3 expression levels was detected in both GBM and skin cancer, an alternative or additional mechanism of transcriptional regulation of RND3 may exist.

Finally, given the critical role of Notch signaling in GBM tumorigenesis, identification of its endogenous inhibitor, RND3, provides a new layer of regulatory complexity to Notch-mediated signaling. The findings also have clinical significance and provide a potential new target for pharmacological manipulation for Notch activation. The assessment of RND3 expression levels in GBM patients could be a new reference biomarker for tumor growth and patient prognosis.

## Supporting Information

Figure S1. The Inhibitory effect of RND3 on glioblastoma cell proliferation was also detected in another glioblastoma cell line, U87. (A) The cell growth rates were assessed under the conditions of overexpression and knockdown of RND3, respectively. (B) Consistent with the changes of the growth rate, the phospho-histone 3 levels were assessed and exhibited a negative correlation with RND3 expression. Data represent means ± SE. *P*-values were from one way ANOVA test. **Figure S2.** The negative regulation of RND3 on Notch signaling was also detected in U87 glioblastoma cells. (A) Forced expression of RND3 inhibited Notch1 target Hes1 transcript (left panel) and protein levels (right panel). (B) Knockdown of RND3 resulted in upregulation of Hes1 transcript (left panel) and protein levels (right panel). (C–D) The luciferase assays showed a negative correlation between RND3 expression and Notch activity. The luciferase activity was driven by CSL-responsive elements. The number at the top of each band represents the average of densitometries from three experiments, normalized by GAPDH. *P* values were from Student's *t*-test. **Figure S3.** Proposed molecular mechanism of RND3 deficiency-mediated glioma cell proliferation and GBM growth. RND3 interacts with Notch transcriptional complex cofactors, NICD, CSL, and Maml1; and facilitates the binding of NICD to Fbw7, which promotes the degradation of NICD, and therefore disassembles the transcriptional complex. The latter prevents hyperactivation of Notch transcriptomes. In the absence of RND3, Notch signaling is enhanced, owing to the extra amount of Notch transcriptional complex formation that facilitates glioma cell proliferation and promotes tumor growth.

**Table S1.** Demographics of patients for the assessment of RND3, HES5, and p-His3 expressions.

**Table S2.** Demographics of patients for the analysis of the relationship among RND3 levels, notch activity, tumor size, and patient survival.
