# Supplementary material for: Downregulation of RND3/RhoE in glioblastoma patients promotes tumorigenesis through augmentation of notch transcriptional complex activity
Source: Cancer Med. 2015 Jun 24;4(9):1404–16. doi: 10.1002/cam4.484 (PMC4567025; doi:10.1002/cam4.484)
Supplement: Figure S1. — The Inhibitory effect of RND3 on glioblastoma cell proliferation was also detected in another glioblastoma cell line, U87. (A) The cell growth rates were assessed under the conditions of overexpression and knockdown of RND3, respectively. (B) Consistent with the changes of the growth rate, the phospho-histone 3 levels were assessed and exhibited a negative correlation with RND3 expression. Data represent means ± SE. P-values were from one way ANOVA test. Figure S2. The negative regulation of RND3 on Notch signaling was also detected in U87 glioblastoma cells. (A) Forced expression of RND3 inhibited Notch1 target Hes1 transcript (left panel) and protein levels (right panel). (B) Knockdown of RND3 resulted in upregulation of Hes1 transcript (left panel) and protein levels (right panel). (C–D) The luciferase assays showed a negative correlation between RND3 expression and Notch activity. The luciferase activity was driven by CSL-responsive elements. The number at the top of each band represents the average of densitometries from three experiments, normalized by GAPDH. P values were from Student's t-test. Figure S3. Proposed molecular mechanism of RND3 deficiency-mediated glioma cell proliferation and GBM growth. RND3 interacts with Notch transcriptional complex cofactors, NICD, CSL, and Maml1; and facilitates the binding of NICD to Fbw7, which promotes the degradation of NICD, and therefore disassembles the transcriptional complex. The latter prevents hyperactivation of Notch transcriptomes. In the absence of RND3, Notch signaling is enhanced, owing to the extra amount of Notch transcriptional complex formation that facilitates glioma cell proliferation and promotes tumor growth. [file cam40004-1404-sd1.pdf]

Supporting Information

Downregulation of RND3/RhoE in Glioblastoma Patients Promotes Tumorigenesis through Augmentation of Notch Transcriptional Complex Activity

Baohui Liu et al.

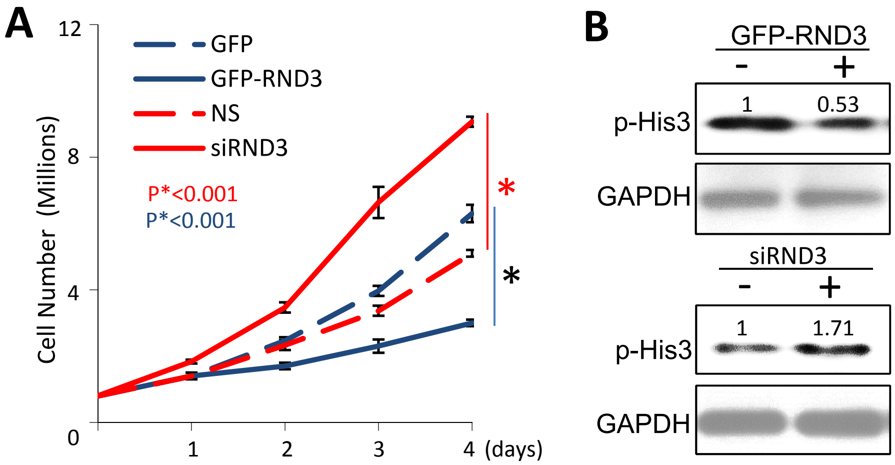

Figure S1. The Inhibitory effect of RND3 on glioblastoma cell proliferation was also detected in another glioblastoma cell line, U87. (A) The cell growth rates were assessed under the conditions of overexpression and knockdown of RND3, respectively. (B) Consistent with the changes of the growth rate, the phospho-histone 3 levels were assessed and exhibited a negative correlation with RND3 expression. Data represent means ± S.E. P values were from one way ANOVA test.

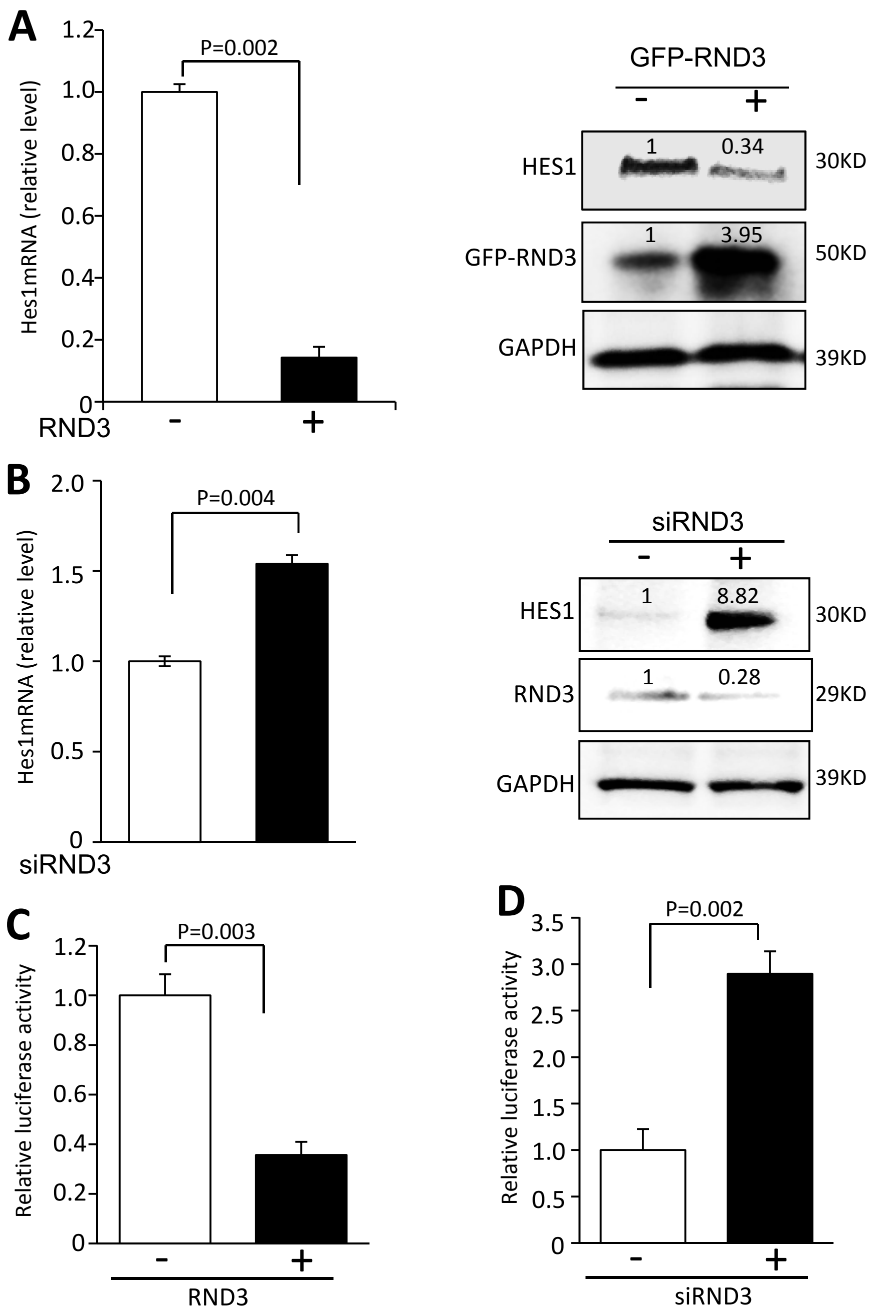

Figure S2. The negative regulation of RND3 on Notch signaling was also detected in U87 glioblastoma cells. (A) Forced expression of RND3 inhibited Notch1 target Hes1 transcript (left panel) and protein levels (right panel). (B) Knockdown of RND3 resulted in upregulation of Hes1 transcript (left panel) and protein levels (right panel). (C-D) The luciferase assays showed a negative correlation between RND3 expression and Notch activity. The luciferase activity was driven by CSL-responsive elements. The number at the top of each band represents the average of densitometries from three experiments, normalized by GAPDH. P values were from Student's *t*-test.

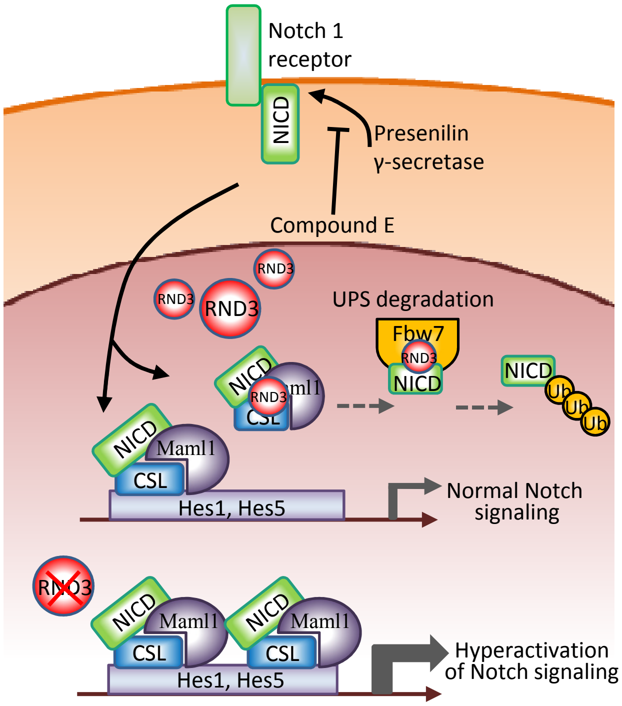

Figure S3. Proposed molecular mechanism of RND3 deficiency-mediated glioma cell proliferation and GBM growth. RND3 interacts with Notch transcriptional complex co-factors, NICD, CSL, and Maml1; and facilitates the binding of NICD to Fbw7, which promotes the degradation of NICD, and therefore disassembles the transcriptional complex. The latter prevents hyperactivation of Notch transcriptomes. In the absence of RND3, Notch signaling is enhanced, owing to the extra amount of Notch transcriptional complex formation that facilitates glioma cell proliferation and promotes tumor growth.
