# Supplementary figures and images for: Downregulation of RND3/RhoE in glioblastoma patients promotes tumorigenesis through augmentation of notch transcriptional complex activity
Source: Cancer Med. 2015 Jun 24;4(9):1404–16. doi: 10.1002/cam4.484 (PMC4567025; doi:10.1002/cam4.484)

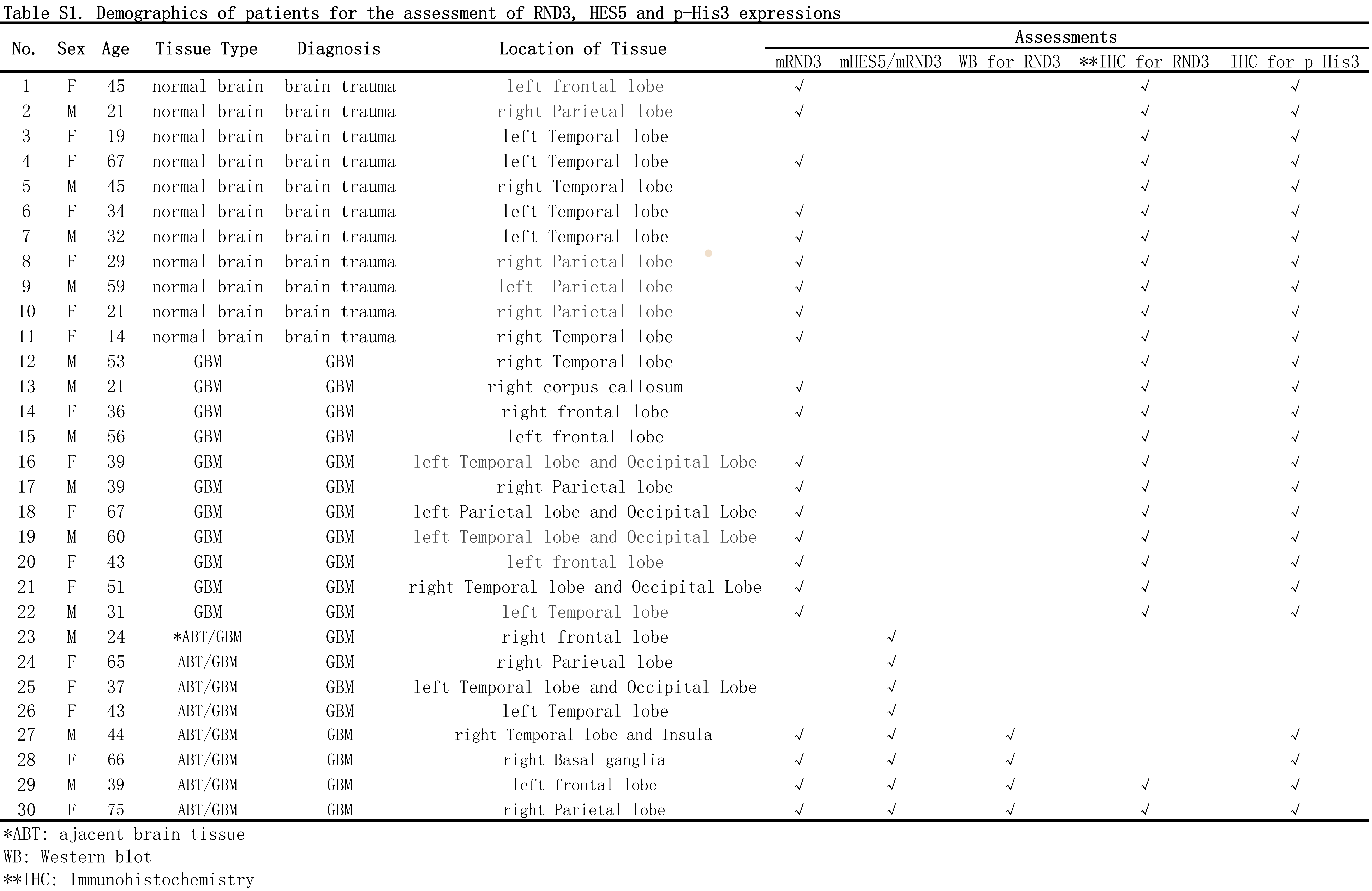

Supplement: Supplementary file 2 — Table S1. Demographics of patients for the assessment of RND3, HES5, and p-His3 expressions. [file cam40004-1404-sd2.tif]

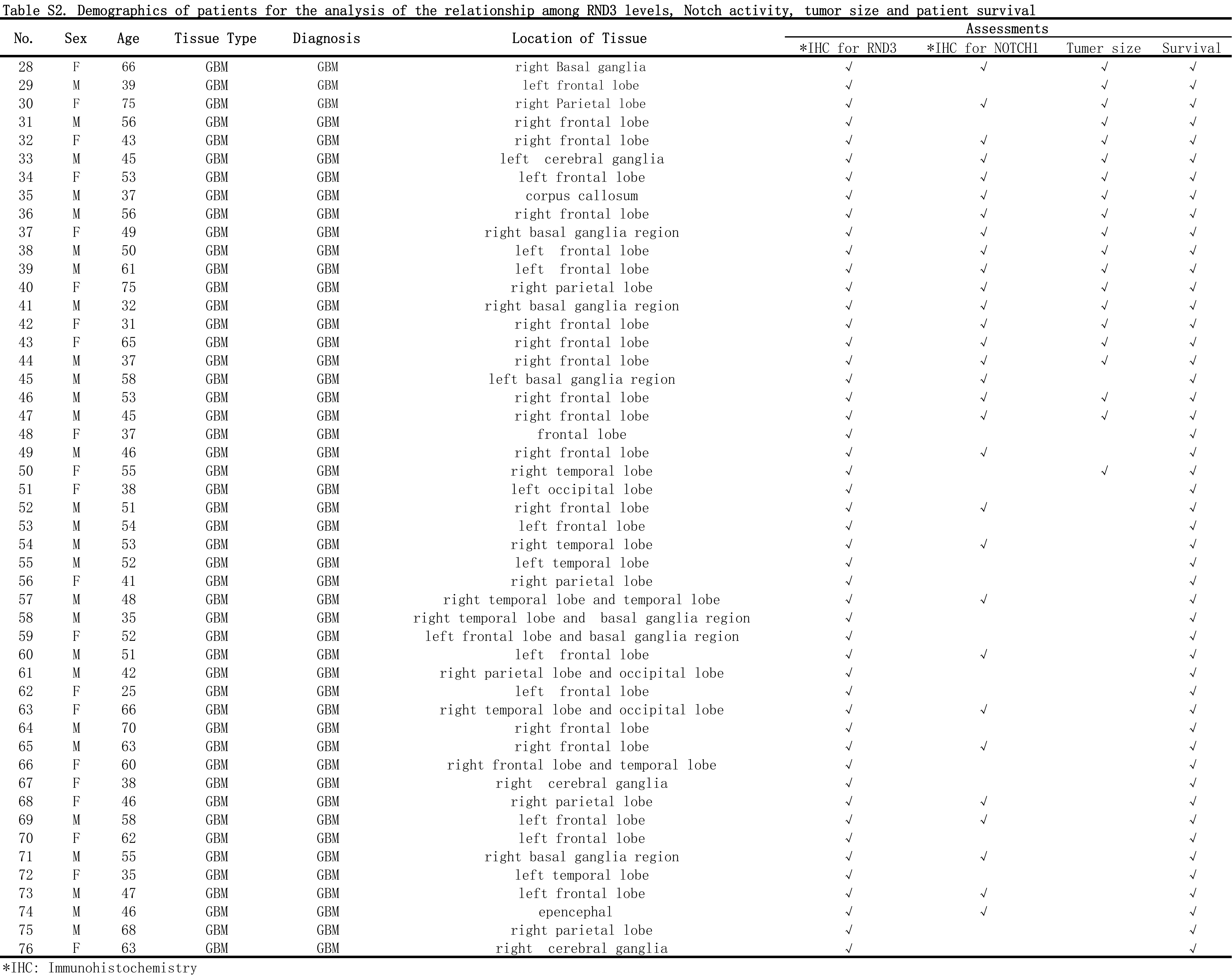

Supplement: Supplementary file 3 — Table S2. Demographics of patients for the analysis of the relationship among RND3 levels, notch activity, tumor size, and patient survival. [file cam40004-1404-sd3.tif]
